# Association between fluoride intake from drinking water and severity of dental fluorosis in Northern and Western Mexico: systematic review and meta-analysis

**DOI:** 10.1186/s12903-024-04472-7

**Published:** 2024-06-19

**Authors:** José Gamarra, David Álvarez-Ordaz, Nelly Molina-Frechero, Leonor Sánchez-Pérez, Alberto Pierdant-Rodriguez, Mario Alberto Isiordia-Espinoza, León Francisco Espinosa-Cristóbal, Marcelo Gómez Palacio-Gastelum, Rogelio González-González, José Salas-Pacheco, Ronell Bologna-Molina

**Affiliations:** 1 Master’s Degree in Dental Sciences, Universidad Autónoma Metropolitana Unidad Xochimilco, Mexico City, Mexico; 2 Medical Sciences Research Institute. Clinical Department, Centro Universitario Los Altos, Universidad de Guadalajara (UdG) Tepatitlán de Morelos, Jalisco, Mexico; 3 Master’s Degree in Dental Sciences, Institute of Biomedical Sciences, Universidad Autónoma Ciudad Juárez, Ciudad Juárez, Chihuahua, 32310 Mexico; 4 Universidad Juarez del Estado de Durango, Durango, Mexico; 5 Universidad de la República, Montevideo, Uruguay

**Keywords:** Fluoride in drinking water, Dental fluorosis, Northern and western Mexico

## Abstract

**Background:**

Dental fluorosis (DF) is caused by excessive exposure to fluoride during odontogenesis and leads to various changes in the development of tooth enamel. Some regions in Mexico are considered endemic fluorosis zones due to the high fluoride content in drinking water. The objective of this study was to perform a systematic review and meta-analysis to identify the association between the concentration of fluoride in drinking water and the severity of dental fluorosis in northern and western Mexico.

**Methods:**

This protocol was registered in the PROSPERO database (ID: CRD42023401519). The search for information was carried out in the PubMed/Medline, Scopus, SpringerLink, and Google Scholar databases between January 2015 and October 2023. The overall relative risk was calculated using the inverse of variance approach with the random effects method. The RoB 2.0 tool was used to construct risk plots.

**Results:**

Eleven articles were analyzed qualitatively, and most of the included studies presented at least one level of DF severity; six articles were analyzed quantitatively, dividing them into two regions. In North region it was observed a higher prevalence of severe TF cases, corresponding to ≥ TF 5 category (4.78) [3.55, 6.42]. In the West region, most of the included studies presented a higher prevalence of less severe cases, corresponding to ≤ TF 4, in comparison with the North region (0.01) [0.00, 0.52], interpreted as a protective effect.

**Conclusion:**

The concentrations of fluorides in drinking water are reportedly high in these regions and are directly related to the severity of dental fluorosis experienced by the inhabitants. In the Northern region exists a major concentration of fluoride in drinking water compared with the Western region as well as a prevalence of higher severity cases of dental fluorosis.

**Supplementary Information:**

The online version contains supplementary material available at 10.1186/s12903-024-04472-7.

## Introduction

Dental fluorosis is caused mainly by excessive fluoride exposure during odontogenesis (a biological process by which teeth form from embryonic cells, grow, and erupt into the mouth), which leads to various changes in tooth enamel development and alters tooth structure (the period from six months to four years is considered to be at greater risk for development) [1–3]. This disease can occur through the ingestion of high fluoride doses (above those established by regulatory standards) and sustained exposure, mostly by drinking water consumption [4].

To perform the diagnostic of dental fluorosis, the Dean index (DI) and the Thlystrup-Fejershow index (TF) can be employed, even though these indexes have differences among them, both could be used at the same time, by granting them a shared numerical value taking into account the enamel affection observed, grouping it in the same grade of affectation [5].

In Mexico, groundwater provides most of the drinking water, and the current geological knowledge indicates that there are some areas where the natural fluoride concentration is high, exceeding the regulatory amount [6]. According to the Mexican normative limit, the maximum fluoride concentration allowed in natural water is 1.5 ppm, and the limit for bottled water is 0.7 ppm [7]. Some regions in Mexico are considered endemic fluorosis zones due to the high fluoride concentrations in drinking water. A recent study has reported that the highest fluoride water consumption in Mexico occurs mainly in the North and West regions of the country [8].

A fluoridation salt program is applied in areas where the concentration of fluoride in water is lower than 0.7 mg/l [9]. This data indicates that these regions are mostly exposed to high fluoride concentrations by drinking water rather than other variables like fluoridated salt, according with some studies 20 million people in those areas are consuming drinking water from supplies with fluoride concentrations that are above the national and international normative, emphasizing states such as San Luis Potosí, Durango, Zacatecas, Jalisco, Chihuahua and Sonora [10, 11].

Considering the existence of different concentrations of fluoride in drinking water in these regions, a lot of cases of dental fluorosis and the scarce evidence reported within the region with major affection, it was proposed to make a review of the literature to determine which region presents a major probability to develop more severe cases of dental fluorosis by associating fluoride intake in drinking water and severity of dental fluorosis.

## Materials and methods

### Research design

Descriptive and retrospective review of science articles published for the elaboration of this systematic review and meta-analysis.

### Protocol and registration

The protocol was registered in the PROSPERO database [12] (ID: CRD42023401519). This study was carried out following the PRISMA guidelines [13].

### Population, exposure, control and outcome. PECO strategy

We used PECO [14] Strategy as follows: P: Habitants living in the Northern and Western region of Mexico, E: Levels of fluoride in drinking water C: Northern and Western regions O: Dental fluorosis severity degrees.

### Eligibility criteria

#### Inclusion criteria

The research included (1) original research articles and full-text articles in English and Spanish; (2) articles that establish a relationship between fluoride intake and dental fluorosis, making special mention of the severity of dental fluorosis in the population of northern and western Mexico; (3) articles that mention the number of participants in the analysis of severity of dental fluorosis; and (4) studies that use the Dean Index (DI) or the Thylstrup and Fejerskov (TF) index to diagnose the severity of dental fluorosis (5) Studies published between January 2015 and August 2023.

#### Exclusion criteria

Review articles, meta-analyses, letters to the editor and original research articles that did not report dental fluorosis or its relation to fluoride intake were excluded because they were not expressly related to the purposes of the research.

### Information sources

The information was searched in the following databases: PubMed/Medline, Scopus, SpringerLink, and Google Scholar.

### Search strategy

Articles in Spanish were used, resulting in 5 articles in this language in the initial search and 85 articles in English. The source of information was exclusively primary. On the initial research 90 articles were found which of them, 11 articles were analyzed for the qualitative study and 6 for the quantitative study.

Keywords were used considering the following medical subject heading (MeSH) guidelines and following the keywords used in the articles having relationship with the aim: “dental fluorosis”, “enamel fluorosis”, “drinking water”, “México”, “potable water”, and “fluoride concentration”. This keywords were combined with the booleans AND, AND/OR as: (dental fluorosis) AND (drinking water); (enamel fluorosis) AND (drinking water); (dental fluorosis) AND (México), (dental fluorosis) AND/OR (drinking water) AND/OR (fluoride concentration).

### Selection process

Two reviewers separately selected the titles and abstracts according to the inclusion criteria previously described. Studies that were selected as relevant were recovered for evaluation of the complete text. Finally, after discussion and agreement of both reviewers, the articles with useful content were chosen.

### Data collection process

Two authors individually extracted the quantitative and qualitative data from the selected articles. Standardized forms were used to facilitate the analysis of the information. A third reviewer was consulted in case of disagreement between the two authors.

### Information data

The data analyzed from each article were collected in Microsoft Excel spreadsheets (2016) in the following order: study authors, year, place, number of participants, fluoride concentration in drinking water, prevalence, and severity of dental fluorosis.

### Methodological quality assessment and risk of bias

To evaluate the methodological quality, the Joanna Briggs Institute (JBI) [15] instrument was used to provide a series of parameters for the methodological evaluation of each article; if the article met the parameters, it was assigned a point to determine the methodological quality.

All the articles included were cross-sectional studies, scores of 1 to 3 were considered low quality, 4 to 6 were considered moderate quality, and 7 to 8 were considered high quality.

The Cochrane Collaboration tool [16] was used to evaluate the risk of bias in different domains, which were classified as low risk, moderate risk, or high risk according to the bias identified. In relation to the results observed in each domain, the overall risk was determined, and the RoB 2.0 [17] tool was used to construct graphs indicating the risk of bias in the individual studies and to analyze the risk of bias in all the studies as a whole.

### Effect measures

The dichotomous outcomes of the population with different percentages of patients according to the fluorosis severity indexes were analyzed, separating the studies into northern and western regions. For the method of analysis, an inverse variance statistical method was used, with a random effects analysis model and an Odds ratio (OR).

### Synthesis methods

Data synthesis was performed with Review Manager 5.4 statistical software, after which the results of the meta-analysis, sensitivity analysis, odds ratio and heterogeneity were calculated. A random effects model was established for this procedure because all the studies were conducted in different populations in which water with different fluoride concentrations was consumed. All outcomes were calculated with a 95% confidence interval (CI).

## Results

### Study selection

**Fig. 1:**
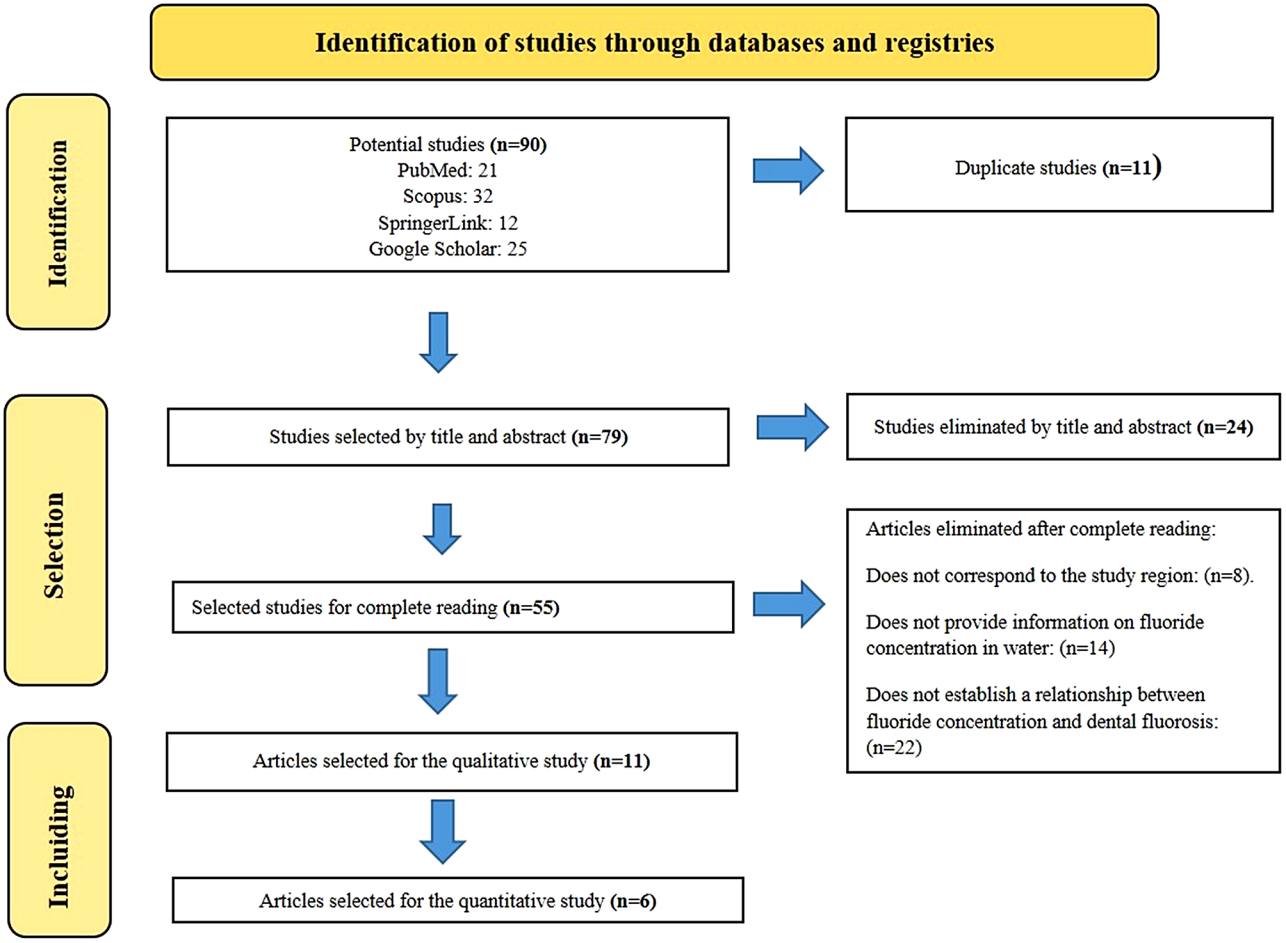
PRISMA flow diagram for the selection of studies included in the systematic review and meta-analysis

Figure 1 shows the article selection process. From an electronic search, 90 articles were registered. First, 11 articles were excluded because they were duplicates.

After reading the title and abstracts, 24 articles were excluded for noncompliance with the eligibility criteria. The other 55 articles were evaluated in full text; 44 additional articles were excluded because they did not correspond to the study region, did not provide information on the concentration of fluoride in water, or did not establish a relationship between fluoride concentration and dental fluorosis.

Finally, 11 articles were selected for the systematic review, and 6 were selected for the meta-analysis; of the 11 articles, 5 were excluded because they did not provide statistical data that met the objective of the present investigation.

### Methodological quality assessment and risk of bias

The Joanna Briggs Institute (JBI) tool [15] was used to assess methodological quality. All the studies evaluated had scores of moderate or high.

Of the 11 articles, 9 had a moderate quality score: Jarquín-Yañez et al. (2015) [18], Escobar García et al. (2015) [19], Aguilar-Díaz et al. (2016) [20], Jarquín-Yañez et al. (2018) [21], Contreras-Espinoza et al. (2018) [22], Tremillo-Maldonado et al. (2020) [23], Duran-Merino et al. (2020) [24], Ontiveros et al. (2020) [25], Farías et al. (2021) [26] and 2 had a high quality score: Molina-Frechero et al. (2017) [6] and González- Dávila et al. (2021) [27].

**Fig. 2:**
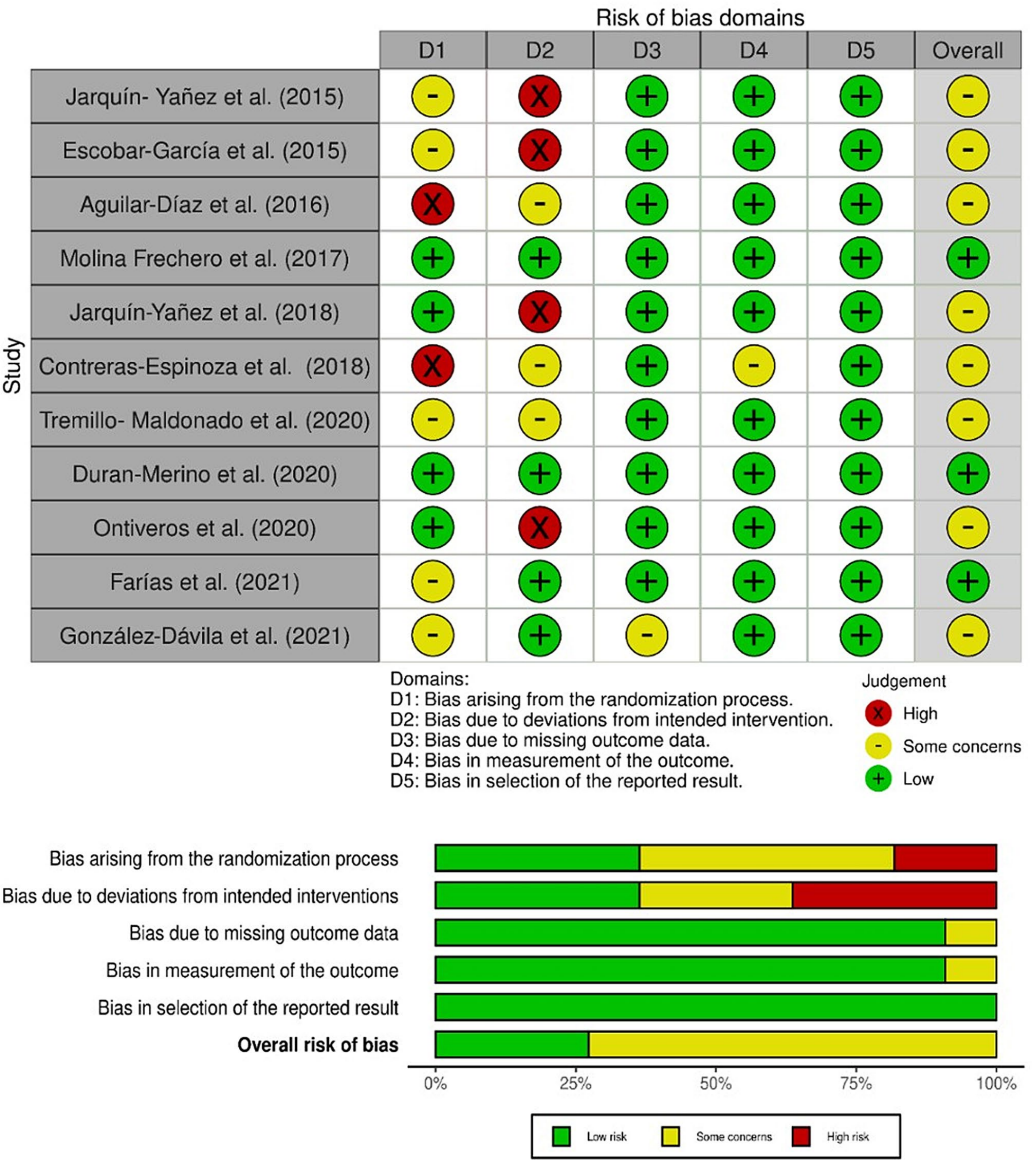
(**a**) Risk of bias for each selected (**b**) article and overall risk of bias

Figure 2 shows the results of the individual assessment of the risk of bias in the selected articles. Eight articles (72.7%) presented a moderate risk of bias, and three articles presented a low risk (27.2%).

### Study characteristics

**Fig. 3:**
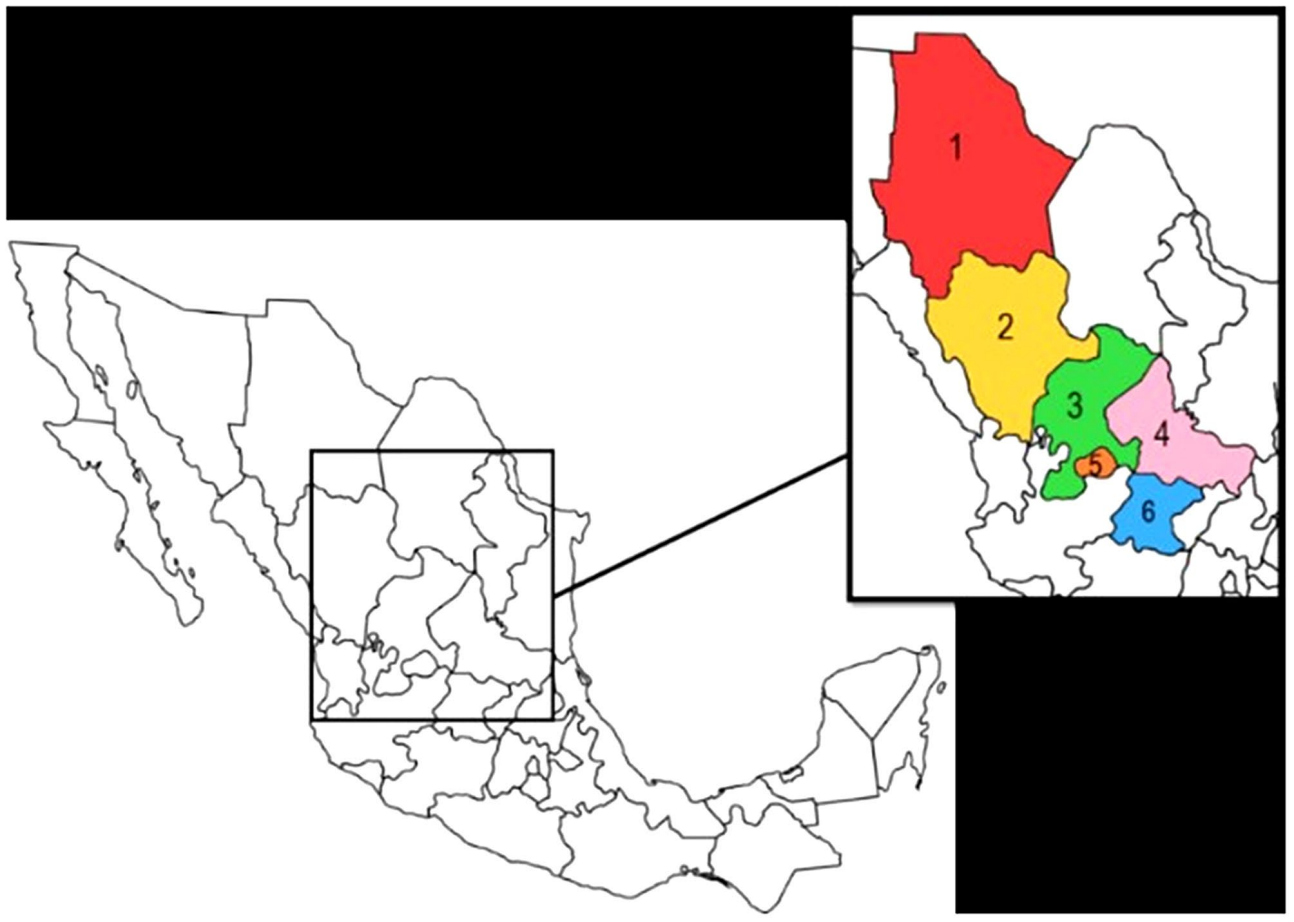
Regions of northern and western Mexico delimiting the areas covered by the study. Northern Regions: (1) Chihuahua, (2) Durango. western Regions: (3) Zacatecas, (4) San Luis Potosí, (5) Aguascalientes, (6) Guanajuato

Eleven articles were included from January 2015 to October 2023, and the eligibility criteria were defined for this investigation, considering the delimited areas. Most of the articles selected (*n* = 11) were from the northern region (*n* = 4) and were distributed as follows: Chihuahua (*n* = 1) and Durango (*n* = 3). The western region (*n* = 7) included the following species: Zacatecas (*n* = 1), San Luis Potosí (*n* = 3), Aguascalientes (*n* = 1), and Guanajuato (*n* = 2) (Fig. 3).

### Quantitative analysis

The level of fluoride in drinking water and the degree of severity of fluorosis were evaluated in 11 studies from different states. Four studies used the Dean index and included normal, questionable, very mild, mild, moderate and severe severity levels. Seven studies used the TF index, with severity levels ranging from 0 to 9. The majority of the population in these studies presented at least one level of fluorosis severity. In addition, in 10 of the 11 studies, the level of fluoride in the water exceeded the limit established in Mexican regulations (Table 1).

**Table 1:** Characteristics of the studies included in the systematic review (*n* = 11)

Author and year of publication	Study site	Study population (*n*)	Degrees of severity of dental fluorosis (Dean or TF index).	Fluoride concentration in water.
Jarquín-Yañez (2015)	San Luis Potosí	(*n* = 111)	TF4-TF5 (*n* = 33) TF6-TF7 (*n* = 50) TF8-TF9 (*n* = 28)	4.13 mg/L
Escobar-García (2015)	San Luis Potosí	(*n* = 83)	TF4-TF5 (*n* = 26) TF6-TF7 (*n* = 35) TF8-TF9 (*n* = 22)	3.9–5.3 mg/L
Aguilar-Díaz (2016)	Guanajuato.	(*n* = 307)	TF0 (*n* = 25) TF1-TF2 (*n* = 54) TF3-TF4 (*n* = 66) TF5-TF6 (*n* = 124) TF7-TF8 (*n* = 36) TF9 (*n* = 2)	4.42 ppm
Molina-Fechero (2017)	Durango	(*n* = 308)	TF2-TF3 (*n* = 99) TF4-TF5(*n* = 172) TF6-TF7 (*n* = 37)	2.51–5.14 ppm
Jarquín Yañez (2018)	San Luis Potosí	(*n* = 230)	TF2-TF5 (*n* = 191) TF6-TF9 (*n* = 39)	2.00–6.00 mg/L
Contreras-Espinoza. (2018)	Zacatecas	(*n* = 207)	Normal (*n* = 66) Questionable (*n* = 75) Very Mild (*n* = 20) Mild (*n* = 21) Moderate (*n* = 21) Severe (*n* = 4)	> 1ppm
Tremillo-Maldonado (2020)	Durango	(*n* = 26)	TF0 (*n* = 1) TF1-TF4 (*n* = 15) TF5-TF9 (*n* = 10)	4–7 ppm
Duran-Merino (2020)	Durango	(*n* = 47)	TF1-TF4 (*n* = 24) TF5-TF9 (*n* = 23)	> 4 ppm
Ontiveros (2020)	Chihuahua	(*n* = 100)	Normal (*n* = 2) Questionable (*n* = 6) Very Mild (*n* = 8) Mild (*n* = 13) Moderate (*n* = 19) Severe (*n* = 52)	2.06–2.74 mg/L
Farías (2021)	Guanajuato	(*n* = 39)	Normal (*n* = 6) Questionable (*n* = 1) Very Mild (*n* = 18) Mild (*n* = 10) Moderate (*n* = 2) Severe (*n* = 2)	3.7–4.9 mg/L
González-Dávila (2021)	Aguascalientes	(*n* = 1052)	Normal (*n* = 596) Mild (*n* = 346) Moderate (*n* = 88) Severe (*n* = 21)	1.16–6.27 ppm

### Quantitative analysis

For the meta-analysis, only 6 articles were included out of the 11 articles previously selected for the systematic review. Although the indices are similar, they are not the same, but for methodological purposes, a categorization of them was carried out to make a comparison between the northern and western regions as follows:

We separated two regions; North and West of Mexico, using both Dean and TF indexes, which are used to determine the severity of the disease and its relationship with fluoride levels in water, were categorized. Two degrees of severity were established: one according to the Dean criteria, from healthy to moderate, corresponding to TF ≤ 4; and one according to the Dean criteria, from severe disease according to the TF index corresponding to TF ≥ 5, with the concentration of fluoride in the water reported by the authors (Table 2).

**Table 2:** Patient characteristics (*n* = 6) included in the meta-analysis

	Author and year of publication	Place and population studied (*n*)	Severity DF ≤ 4	Severity DF ≥ 5	Fluoride concentration in water.
Northern region	Molina-Frechero (2017)	Ciudad de Durango (*n* = 308)	99 (32.14%)	209 (67.86%)	3.84 ppm
	Ontiveros (2020)	Chihuahua (*n* = 100)	29 (29%)	71 (71%)	2.29 ppm
Western region	Aguilar-Díaz (2016)	Guanajuato (*n* = 307)	118 (38.43%)	189 (61.57%)	4.42 ppm
	Contreras-Espinoza (2018)	Zacatecas (*n* = 207)	182 (87.92%)	25 (12.08%)	1ppm
	Farías (2021)	Guanajuato (*n* = 78)	72 (92.30%)	6 (7.70%)	2.1 ppm
	González-Dávila (2021)	Municipio Aguascalientes (*n* = 625)	613 (98.08%)	12 (1.92%	1.90 ppm
		Municipio Calvillo y Rincón de Romos (*n* = 152)	148 (97.36%)	4 (2.64%)	1.25 ppm

**Fig. 4:**
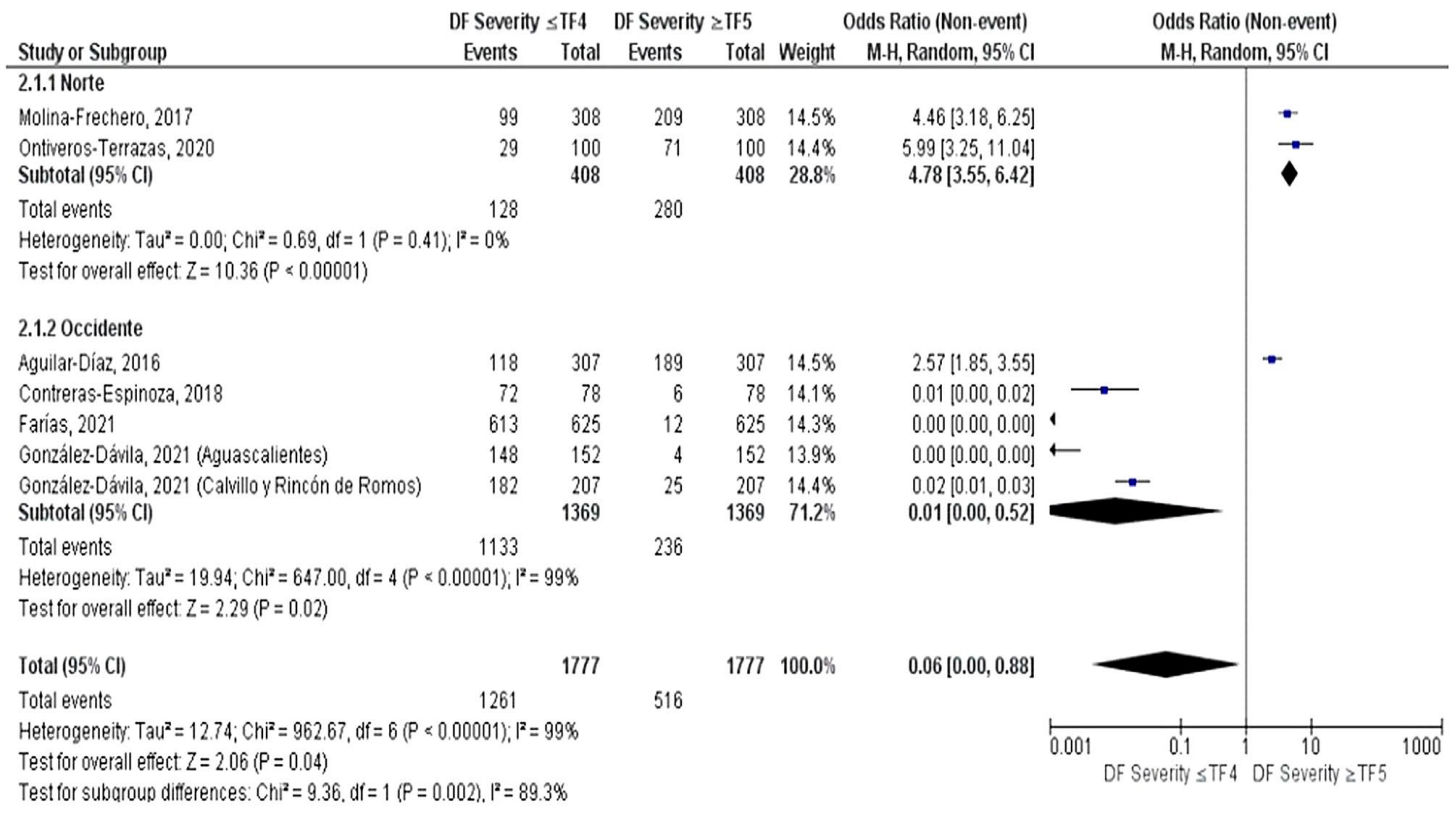
Forest plot of the severity of DF in the northern and western region

In the quantitative analysis, a subgroup meta-analysis was produced, taking into the categorization described previously, we decided to use a random effects model due to the differences of the included studies, also we used an Odds Ratio statistical measure to compare the total events between both regions because of the design of the selected studies, which were cross-sectional studies.

Both regions were compared considering the number of presented dental fluorosis cases. In North region it was observed a higher prevalence of severe TF cases, corresponding to ≥ TF 5 category (4.78) [3.55, 6.42], the studies included in these regions presented a I^2^ value of 0% indicating a low heterogeneity among both studies, this value may represent the existing methodological similarities between these two studies (Fig. 4).

**Fig. 5:**
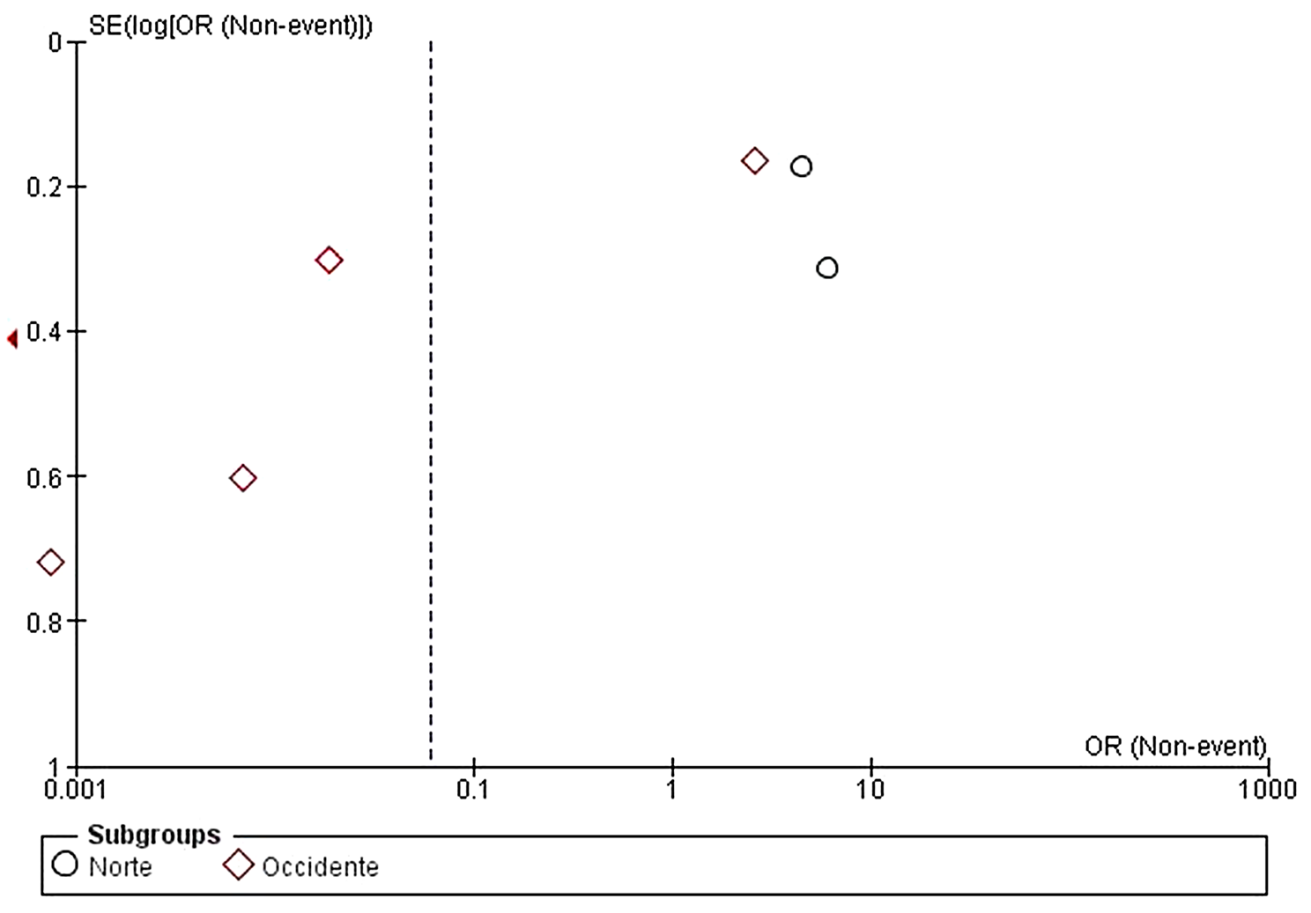
Funnel plot for the measurement of publication bias in the northern and western

In the West region, most of the included studies presented a higher prevalence of less severe cases, corresponding to ≤ TF 4, in comparison with the North region (0.01) [0.00,0.52] interpreted as a protective effect, an I^2^ value of 99%, observing a high heterogeneity, this is shown in a forest plot in Fig. 5, where the reported data by Farías and González Dávila are not found in the confidence intervals, this may be due to the big differences between the reported events and it´s samples sizes (Fig. 4).

Even though we could interpret in the forest plot that there is a higher probability in Northern region to carry more severe cases of dental fluorosis, we should take into account that in the total analysis of both groups it is reported a confidence interval of 0.06 [0.00,0.88], data that is not conclusive, as well as the heterogeneity value of 99% which is considered high, the big difference among the sample size in each study, quantity of reported events and the mentioned categorization made for this study as shown in the funnel plot (Fig. 5), where a big asymmetric dispersion of studies can be observed, specially in the Western region studies, which reflects what was said previously.

## Discussion

The objective of the present study was to identify the association between drinking water fluoride concentration and dental fluorosis severity grades in populations from the North and West Zones of Mexico.

Dental fluorosis can be described as a multifactorial pathology [28] that depends on each characteristic, including genetic, environmental, nutritional, and/or socioeconomic characteristics, such as the main method of water consumption. Most of the articles that were included in the systematic review (*n* = 11) reported fluoride concentrations in drinking water higher than the limit established by the Mexican standard (1.5 mg/L) [7]. Only one study, carried out in Zacatecas, reported a concentration of 1 ppm, which corresponds to 1 mg/L, and the entire study population, in all the articles, presented some degree of severity of DF.

Several studies have reported that there may be variations in the degree of severity of dental fluorosis according to genetic factors, since there are areas with high fluoride content in water where the population presents clinical manifestations of the disease similar to those of populations that present lower concentrations of fluoride in water. This may be due to the association of gene expression with the severity of dental fluorosis [18–21, 23, 24].

For the meta-analysis, 6 articles that fulfilled the quantitative criteria were included. Two groups were established and divided into North and West regions, following the standardization of the DF ≤ TF4 and DF ≥ TF5 grades to categorize the severity grades.

The analysis brings us first to the northern region, where Molina Frechero established, as Ontiveros et al. did, that all recollected water samples exceeded the national parameters [7] and that all the studied populations presented some dental fluorosis grade.

Along with the DF severity, Molina Frechero [6] reported that 71% of the population had a severe dental fluorosis grade (≤ TF5), and all of the participants had been residing in this place since birth. This finding is important and matches the findings of Ontiveros [25]. These findings indicate that 92% of the population in the four studied localities presented any dental fluorosis grade, the most remarkable fact was that 100% of dental fluorosis incidence was observed in the age group of 31 to 40 years. This contrast must be analyzed since a person exposed to high fluoride concentrations, mainly because of drinking water for an extended period, such as since birth, is more likely to develop DF.

Farias [26] from the West Region, where the prevalence of dental fluorosis is 80% in children aged 6 to 14 years, suggests that exposure to fluoride at young ages promotes the development of this pathology.

Ontiveros [25] mentioned that high fluoride concentrations in drinking water represents a public health problem due to the health consequences of those who are exposed. Aguilar Diaz [21] reported in Guanajuato State, which belongs to the western region, one of the highest fluoride concentrations, 4.42 ppm, which is closely linked to 91.1% of the studied population presenting any dental fluorosis grade. Despite reports of a very high fluoride concentration, only 61.6% of this population exhibited severe disease (≤ TF5). Given that a greater percentage of severe affectation due to the high fluoride concentration in water could be expected, the same was shown in the Farías [26] study, which reported a lower fluoride concentration in comparison with the reports from Aguilar-Diaz [20]. 82% of the population has any dental fluorosis grade, but 45% of the population has mild fluorosis; as mentioned by the author, only 2% of the population consumes well and/or tap water, therefore, the population is less exposed, which coincides with the findings of González-Dávila [27] in Aguascalientes, a zone with high fluoride concentration in drinking water and dental fluorosis; however, the author established that, while being an endemic zone, only 9.9% of the participants consumed tap water, which could explain why high water fluoride concentrations but low populations with severe dental fluorosis grades were reported. As shown in the research by Contreras-Espinoza [22] in Zacatecas, given that a DF incidence ≥ TF4 was mentioned as an endemic zone, this information could be due to the characteristics of the studied population, such as its socioeconomic level. Only a 1 ppm fluoride concentration is mentioned, as Gutierrez [29] reports that, according to the data from the National Water Commission, in this region, individuals between the ages 2017 and 2019 were found to consume a fluoride water concentration 114 times higher than the permitted limit, oscillatory values ranging from 0.20 to 22.29 mg/L.

In other studies conducted in other Mexican regions, as evidenced in this study, drinking water fluoride concentrations were reported to be above certain limits [30–33] but any of these studies had the several cases of dental fluorosis like the reported in the North and Western region. Severe dental fluorosis is evident in North Region states, possibly because the well water from this region has a high prevalence of minerals considered toxic, such as fluoride and arsenic, since well excavation for water extraction increases mineral concentrations; even bottled-water fluoride concentrations should be taken into account, as De la Cruz Cardozo [34] mentioned, where it is evidenced that North Region states possessed higher fluoride concentrations than the normative limit in this kind of water. According to studies in the western region, a low percentage of the settlers in the included zones consumed tap water and/or well water; therefore, they were less exposed to these high fluoride concentrations, which explains the lower severity grades. Due to these factors, the quantitative analysis revealed a greater positive tendency in the northern region (2.19 [95% CI 1.87, 2.56]) than in the western region (0.21 [95% CI 0.18, 0.24]).

All the studies included in the systematic review and meta-analysis reported that the studied population had any dental fluorosis severity grade or high drinking water fluoride concentration; however, other variables, such as geographic conditions of these zones, latitude, height, and drinking water origin, especially in deep-well zones or volcanic locations, must be considered because of the high mineral concentrations [35–37].

The collected data revealed that the consumption of water with high fluoride concentrations and different dental fluorosis severity grades, mainly severe grade, are directly related, but the water fluoride concentration cannot be considered the only variable [38, 39]. A deeper search is needed to evaluate other variables which could participate in the development of the dental fluorosis, and determinate if those variables can act together to aggravate the vulnerability of this population. The use of fluoridated toothpaste, even though development of dental fluorosis is mostly related to the fluoride intake and not in the topic use, can increase the risk to develop more severe grades of dental fluorosis.

Within the strengths of the present research, it is necessary to be mentioned that it’s the first review to determinate the region which has a higher association within the principal variable, fluoride intake by drinking water, and the grades of dental fluorosis severity in two endemic areas.

About the limitations, it could be mentioned the aggrupation of the indexes made to categorize the grades of the severity of dental fluorosis, given the differences in both indexes, also heterogeneity of the selected studies must be considered because some zones report high fluoride concentrations in water, but the size of the sample is potentially insufficient and cannot verify the real problem, the lack of the longitudinal studies in the area as the missing criteria concerning the valuation of the origin of water ingestion are missing because most of the population of the western zone does not drink tap water.

## Conclusions

The present investigation showed that the northern and western regions of the Mexican Republic manage concentrations of fluoride in water above the standard established by the Mexican Government. However, it was observed that in the Northern region exists an elevated concentration of fluoride in drinking water compared with the Western region as well as a prevalence of higher severity cases of dental fluorosis, which indicates a major positive tendency in the association between fluoride intake from drinking water and the severity of dental fluorosis. Studies with similar characteristics should be carried out, covering other variables that could lead to greater retention of fluoride in the body.

### Electronic supplementary material

Below is the link to the electronic supplementary material.

Supplementary Material 1

## Data Availability

Datasets generated during and/or analyzed during the present study may be requested from the corresponding author.
