# Supplementary material for: Association between fluoride intake from drinking water and severity of dental fluorosis in Northern and Western Mexico: systematic review and meta-analysis
Source: BMC Oral Health. 2024 Jun 19;24:708. doi: 10.1186/s12903-024-04472-7 (PMC11186227; doi:10.1186/s12903-024-04472-7)
Supplement: Supplementary file 1 — Supplementary Material 1 [file 12903_2024_4472_MOESM1_ESM.pdf]

Appendix 1. Evaluación de la calidad metodológica mediante la lista de comprobación del JBI.

Artículos	Respuesta									
	**1**	**2**	**3**	**4**	**5**	**6**	**7**	**8**	**Puntaje**	**Calidad**
	**Estudios transversales**									
Jarquín- Yañez et al. (2015)[16]	Y	Y	Y	Y	U	U	Y	Y	6	Moderada
Escobar-García et al. (2015)[17]	Y	Y	Y	Y	N	N	Y	Y	6	Moderada
Aguilar-Díaz et al. (2016)[18]	Y	U	Y	Y	N	N	Y	Y	5	Moderada
Molina Frechero et al. (2017)[5]	Y	Y	Y	Y	Y	Y	N	Y	7	Alta
Jarquín-Yañez et al. (2018)[19]	Y	Y	Y	Y	N	N	Y	Y	6	Moderada
Contreras-Espinoza et al. (2018)[20]	N	Y	Y	Y	N	N	Y	N	4	Moderada
Tremillo- Maldonado et al. (2020)[21]	Y	N	Y	Y	N	N	Y	Y	5	Moderada
Duran-Merino et al. (2020)[22]	Y	N	Y	Y	N	N	Y	Y	5	Moderada
Ontiveros et al. (2020)[23]	Y	Y	Y	Y	Y	N	N	Y	6	Moderada
Farías et al. (2021)[24]	Y	Y	Y	Y	N	N	Y	Y	6	Moderada
González-Dávila et al. (2021)[25]	Y	Y	Y	Y	Y	Y	Y	Y	8	Alta

Y: yes; N: no; U: unclear.
